# The College of Nursing (Limited by Guarantee)

**Published:** 1921-02-12

**Authors:** 


### THE COLLEGE OF NURSING

**(Limited by Guarantee.)**

### What the Local Centres are Doing.

### BIRMINGHAM THREE COUNTIES CENTRE.

(Hon. Secretary, Mrs. M. Glegg, 84 Hagley Road.)

The Need for Organisation.

On Tuesday, February 8, in the Lecture Theatre of the General Hospital, Birmingham (by kind permission of the Governors), Mr. W. Benwell gave an interesting and instructive address on "The Organisation of the Middle and Professional Classes, with Special Reference to the Nursing Services." Mr. Benwell traced rapidly the growth of political power amongst the labour classes as a result of their progress in organisation, and he emphasised the fact that Labour is using its political power as a lever for the betterment of industrial conditions. He urged upon his audience the necessity for co-ordination, since the nursing profession is not even collectively organised, and advised the members to endeavour to affiliate with the National Federation of Professional Workers, and benefit by their experience and advice in framing the first principles of their organisation. A national federation of nurses and health visitors affiliated with the British Medical Association would, he said, be sufficiently powerful to bring effectual pressure to bear on the Government with reference to all matters relating to health. In conjunction with other professional organisations such a body should become a powerful political factor in the State.

Payment of Subscriptions.

All members who have not paid their subscriptions are reminded that the Hon. Treasurer, Mrs. Boeddicker, Ellerslie, Ascot Road, Moseley, will be glad to receive them at the earliest possible date.

### THE CORNWALL CENTRE AT TRURO.

(Hon. Secretary, Miss Jordan, Cornwall Convalescent Home, Perranporth.)

At a well-attended meeting, held at the Royal Cornwall Infirmary, Truro, the members of the above Centre and guests listened to a most interesting lecture by Dr. G. F. Burnell on "The Nervous Element in Disease." Miss Bessie Chaff presided. Afterwards there was a meeting of members only, at which the scale of salaries recently recommended by the Council of the College of Nursing was discussed, and the following resolution, proposed by Miss H. M. Brown and seconded by Miss Riden, was carried unanimously, with a view to its circulation to all Chairmen of Committees employing trained nurses in the county: "That this Centre of the College of Nursing begs to draw the attention of your Committee to the scale of salaries recommended by the Council of the College, as set forth under their letter of December 1920, and trusts that your Committee may be able to give effect to this scale for all trained nurses employed on your staff."

A letter from the London Centre with regard to the nomination of candidates for the forthcoming Council election was then discussed, and there was a strongly-expressed opinion that the south-western area should be represented on the Council. It was decided to ask Miss Volta Billing, R.R.C., to accept nomination, and to ask the help of the Plymouth and District and Torquay Centres with a view to securing her election. Miss Billing has consented to be nominated.

### EDINBURGH CENTRE.

(Hon. Secretary, Miss Cathcart, The Elms, Whitehouse Loan.)

Wednesday, February 16, 3.30 p.m., Lecture on "The Nature and Treatment of Functional Nervous Disorders, particularly in Relation to those Arising during the late War," in the Nurses' Club, 8 Drumsheugh Gardens, by Arthur Taylor, Esq., M.D., F.R.C.P.E. (Medical Superintendent, Craigend Neurasthenic Hospital).

### NORFOLK AND NORWICH CENTRE.

(Hon. Secretary, Miss V. A. Rutter, Nurses' Home, 43 Newmarket Road.)

Thursday, February 17, 8 p.m.—Lecture at the Jenny Lind Hospital (by kind permission), on "The Commoner Diseases of the Ear," by N. S. Carruthers, Esq., F.R.C.S. Members free; non-members will be welcome; tickets, 1s.

### MEETING AT AYR: CENTRE TO BE FORMED.

Miss Pike, Secretary to the Scottish Board of the College of Nursing, addressed a representative meeting of the College members and other nurses, held in the County Hospital, Ayr, on Friday of last week on the work of the College of Nursing and the benefits of Local Centres. She outlined the policy of the College, pointed out what had already been accomplished by this body, and spoke of the importance and work of Local Centres.

After some discussion the Chairman, Miss Crichton, matron of the hospital, proposed the formation of a Local Centre for Ayr and the neighbourhood. This was carried unanimously, and it was resolved that another meeting be called at an early date to give further consideration to details in connection with the project.

